# Supplementary figures and images for: Gametophytic self-incompatibility in Andean capuli (Prunus serotina subsp. capuli): allelic diversity at the S-RNase locus influences normal pollen-tube formation during fertilization
Source: PeerJ. 2020 Aug 31;8:e9597. doi: 10.7717/peerj.9597 (PMC7469932; doi:10.7717/peerj.9597)

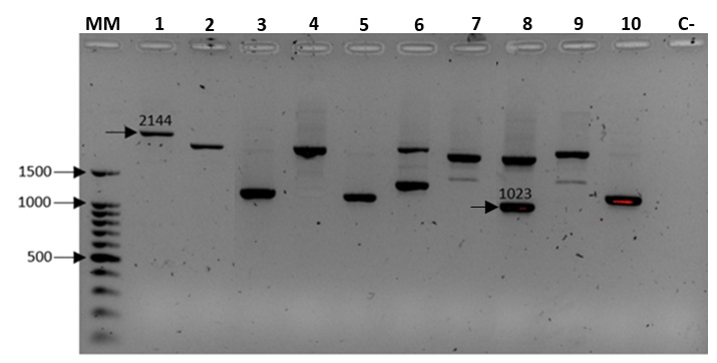

Supplement: Figure S1 — 10 of the 15 amplified accessions are shown in this gel. Lanes are: MM Ladder 100bp Promega, 1. H25, 2. Azu15, 3. Can11, 4. Pic19, 5. Car7, 6. Car3, 7. Can9, 8. Pic023, 9. Can022, 10. Car011. C- Negative Control. The size range of the amplicons is between 2,144 and 1,023 bp [file peerj-08-9597-s001.png]

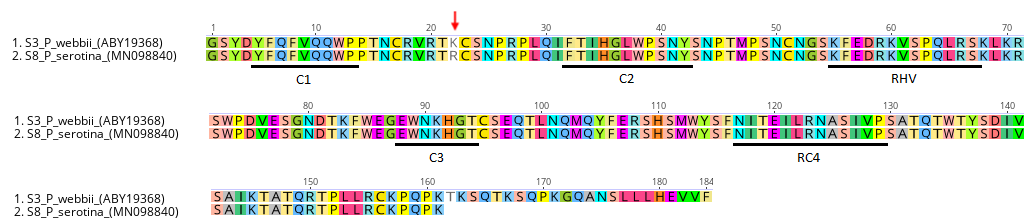

Supplement: Figure S2 — Sequences are 99.38% identical. The conservative amino acid substitution of an arginine (R) by a lysine (K) located between C2 and C3 is pointed out with an arrow [file peerj-08-9597-s002.png]
