# Supplementary material for: Gametophytic self-incompatibility in Andean capuli (Prunus serotina subsp. capuli): allelic diversity at the S-RNase locus influences normal pollen-tube formation during fertilization
Source: PeerJ. 2020 Aug 31;8:e9597. doi: 10.7717/peerj.9597 (PMC7469932; doi:10.7717/peerj.9597)
Supplement: Table S1 [file peerj-08-9597-s003.docx]

| **Code** | **Collection Date** | **Province** | **Coordinates (Decimal Degrees)** | **Altitude** |
| --- | --- | --- | --- | --- |
| AZU15 | 4/3/2012 | Azuay | -2.977750, -78.816669 | 2612 |
| PIC2 | 11/2/2012 | Pichincha | -0.183386, -78.480083 | 2811 |
| PIC19 | 14/4/2012 | Pichincha | 0.046194, -78.229361 | 2889 |
| PIC23 | 14/4/2012 | Pichincha | 0.070833, -78.135250 | 2841 |
| IMB11 | 9/9/2010 | Imbabura | 0.188730, -78.225430 | 2778 |
| H14 | 31/3/2011 | Chimborazo | -1.390800, -78.421470 | 2944 |
| H25 | 31/3/2011 | Chimborazo | -1.370790, -78.393700 | 2902 |
| CAN9 | 3/3/2012 | Cañar | -2.703000, -78.890444 | 2733 |
| CAN11 | 3/3/2012 | Cañar | -2.699250, -78.891556 | 2756 |
| CAN22 | 3/3/2012 | Cañar | -2.761111, -78.857944 | 2526 |
| CAR3 | 25/5/2010 | Carchi | 0.353880, -77.592800 | 3006 |
| CAR5 | 25/5/2010 | Carchi | 0.370170, -77.563500 | 2998 |
| CAR11 | 25/5/2010 | Carchi | 0.300540, -77.541250 | 2621 |
| CAR12 | 25/5/2010 | Carchi | 0.303520, -77.534710 | 2621 |
