# Supplementary material for: Gametophytic self-incompatibility in Andean capuli (Prunus serotina subsp. capuli): allelic diversity at the S-RNase locus influences normal pollen-tube formation during fertilization
Source: PeerJ. 2020 Aug 31;8:e9597. doi: 10.7717/peerj.9597 (PMC7469932; doi:10.7717/peerj.9597)
Supplement: Table S2 — For alleles S2, S6 and S11 only the Intron I sequence could be retrieved and submitted. [file peerj-08-9597-s004.docx]

| ***P. serotina S*-alleles** | **GenBank Accession Number** | **Sequence Submitted** |
| --- | --- | --- |
| **S_1_** | MN098833 | Full length |
| **S_2_** | MN098834 | Intron I |
| **S_3_** | MN098835 | Full length |
| **S_4_** | MN098836 | Full length |
| **S_5_** | MN098837 | Full length |
| **S_6_** | MN098838 | Intron I |
| **S_7_** | MN098839 | Full length |
| **S_8_** | MN098840 | Full length |
| **S_9_** | MN098841 | Full length |
| **S_10_** | MN098842 | Full length |
| **S_11_** | MN098843 | Intron I |
| **S_12_** | MN098844 | Full length |
| **S_13_** | MN098845 | Full length |
| **S_14_** | MN098846 | Full length |
