# Supplementary material for: Gametophytic self-incompatibility in Andean capuli (Prunus serotina subsp. capuli): allelic diversity at the S-RNase locus influences normal pollen-tube formation during fertilization
Source: PeerJ. 2020 Aug 31;8:e9597. doi: 10.7717/peerj.9597 (PMC7469932; doi:10.7717/peerj.9597)
Supplement: Table S3 — The Pistil number column indicates the number of pistils analyzed for each of the 8 crosses performed. Pollen donor and Pollen receptor columns indicate the number of the trees employed for the cross. The S allelic composition of each tree is indicated in parenthesis. The Cross type column indicates the expected outcome of the cross according to the S-allelic composition of the parental trees. Self-pollinations are considered incompatible crosses. [file peerj-08-9597-s005.docx]

|  |  |  |  | **Style** | | |  |  |
| --- | --- | --- | --- | --- | --- | --- | --- | --- |
| **Pistil number** | **Cross type** | **Pollen donor** | **Pollen receptor** | **Upper third** | **Middle third** | **Bottom third** | **Total number of pollen tubes in the pistil** | **Global percentage of pollen tubes reaching the lower third of the style** |
| 1 | Compatible | 17 (S1, S4) | 1 (S8, S19) | 140 | 46 | 10 | 196 | 5,42% |
| 2 | Compatible | 17 (S1, S4) | 1 (S8, S19) | 172 | 34 | 6 | 212 |  |
| 3 | Compatible | 17 (S1, S4) | 1 (S8, S19) | 40 | 10 | 3 | 53 |  |
| 4 | Compatible | 17 (S1, S4) | 1 (S8, S19) | 44 | 6 | 4 | 54 |  |
| 5 | Compatible | 17 (S1, S4) | 1 (S8, S19) | 40 | 10 | 4 | 54 |  |
| 6 | Compatible | 17 (S1, S4) | 1 (S8, S19) | 64 | 18 | 8 | 90 |  |
| 7 | Compatible | 17 (S1, S4) | 1 (S8, S19) | 44 | 12 | 4 | 60 |  |
| 1 | Compatible | 14 (S20, S21) | 15 (S10, S6) | 80 | 18 | 5 | 103 | 4,28% |
| 2 | Compatible | 14 (S20, S21) | 15 (S10, S6) | 100 | 18 | 6 | 124 |  |
| 3 | Compatible | 14 (S20, S21) | 15 (S10, S6) | 32 | 8 | 4 | 44 |  |
| 4 | Compatible | 14 (S20, S21) | 15 (S10, S6) | 120 | 10 | 4 | 134 |  |
| 5 | Compatible | 14 (S20, S21) | 15 (S10, S6) | 128 | 12 | 5 | 145 |  |
| 6 | Compatible | 14 (S20, S21) | 15 (S10, S6) | 24 | 10 | 1 | 35 |  |
| 7 | Compatible | 14 (S20, S21) | 15 (S10, S6) | 20 | 2 | 1 | 23 |  |
| 8 | Compatible | 14 (S20, S21) | 15 (S10, S6) | 20 | 2 | 1 | 23 |  |
| 9 | Compatible | 14 (S20, S21) | 15 (S10, S6) | 20 | 2 | 1 | 23 |  |
| 10 | Compatible | 14 (S20, S21) | 15 (S10, S6) | 20 | 2 | 1 | 23 |  |
| 1 | Self-pollination | 17 (S1, S4) | 17 (S1, S4) | 80 | 16 | 5 | 101 | 3,32% |
| 2 | Self-pollination | 17 (S1, S4) | 17 (S1, S4) | 60 | 14 | 3 | 77 |  |
| 3 | Self-pollination | 17 (S1, S4) | 17 (S1, S4) | 100 | 20 | 4 | 124 |  |
| 4 | Self-pollination | 17 (S1, S4) | 17 (S1, S4) | 32 | 4 | 5 | 41 |  |
| 5 | Self-pollination | 17 (S1, S4) | 17 (S1, S4) | 76 | 28 | 2 | 106 |  |
| 6 | Self-pollination | 17 (S1, S4) | 17 (S1, S4) | 60 | 28 | 2 | 90 |  |
| 7 | Self-pollination | 17 (S1, S4) | 17 (S1, S4) | 24 | 28 | 0 | 52 |  |
| 8 | Self-pollination | 17 (S1, S4) | 17 (S1, S4) | 40 | 2 | 0 | 42 |  |
| 1 | Incompatible | 22 (S1, S4) | 17 (S1, S4) | 84 | 22 | 6 | 112 | 3,41% |
| 2 | Incompatible | 22 (S1, S4) | 17 (S1, S4) | 76 | 32 | 4 | 112 |  |
| 3 | Incompatible | 22 (S1, S4) | 17 (S1, S4) | 88 | 24 | 7 | 119 |  |
| 4 | Incompatible | 22 (S1, S4) | 17 (S1, S4) | 112 | 16 | 11 | 139 |  |
| 5 | Incompatible | 22 (S1, S4) | 17 (S1, S4) | 124 | 28 | 4 | 156 |  |
| 6 | Incompatible | 22 (S1, S4) | 17 (S1, S4) | 120 | 24 | 3 | 147 |  |
| 7 | Incompatible | 22 (S1, S4) | 17 (S1, S4) | 32 | 10 | 2 | 44 |  |
| 8 | Incompatible | 22 (S1, S4) | 17 (S1, S4) | 140 | 10 | 2 | 152 |  |
| 9 | Incompatible | 22 (S1, S4) | 17 (S1, S4) | 48 | 12 | 1 | 61 |  |
| 10 | Incompatible | 22 (S1, S4) | 17 (S1, S4) | 20 | 4 | 1 | 25 |  |
| 11 | Incompatible | 22 (S1, S4) | 17 (S1, S4) | 44 | 16 | 8 | 68 |  |
| 12 | Incompatible | 22 (S1, S4) | 17 (S1, S4) | 60 | 12 | 5 | 77 |  |
| 13 | Incompatible | 22 (S1, S4) | 17 (S1, S4) | 48 | 20 | 0 | 68 |  |
| 14 | Incompatible | 22 (S1, S4) | 17 (S1, S4) | 36 | 0 | 0 | 36 |  |
| 15 | Incompatible | 22 (S1, S4) | 17 (S1, S4) | 40 | 6 | 0 | 46 |  |
| 16 | Incompatible | 22 (S1, S4) | 17 (S1, S4) | 52 | 2 | 0 | 54 |  |
| 17 | Incompatible | 22 (S1, S4) | 17 (S1, S4) | 96 | 0 | 0 | 96 |  |
| 18 | Incompatible | 22 (S1, S4) | 17 (S1, S4) | 68 | 2 | 0 | 70 |  |
| 1 | Self-pollination | 1 (S8, S19) | 1 (S8, S19) | 104 | 0 | 3 | 107 | 2,03% |
| 2 | Self-pollination | 1 (S8, S19) | 1 (S8, S19) | 64 | 0 | 2 | 66 |  |
| 3 | Self-pollination | 1 (S8, S19) | 1 (S8, S19) | 44 | 18 | 0 | 62 |  |
| 4 | Self-pollination | 1 (S8, S19) | 1 (S8, S19) | 112 | 34 | 0 | 146 |  |
| 5 | Self-pollination | 1 (S8, S19) | 1 (S8, S19) | 52 | 10 | 0 | 62 |  |
| 6 | Self-pollination | 1 (S8, S19) | 1 (S8, S19) | 16 | 2 | 1 | 19 |  |
| 7 | Self-pollination | 1 (S8, S19) | 1 (S8, S19) | 16 | 12 | 4 | 32 |  |
| 8 | Self-pollination | 1 (S8, S19) | 1 (S8, S19) | 68 | 4 | 2 | 74 |  |
| 9 | Self-pollination | 1 (S8, S19) | 1 (S8, S19) | 8 | 8 | 0 | 16 |  |
| 10 | Self-pollination | 1 (S8, S19) | 1 (S8, S19) | 4 | 2 | 0 | 6 |  |
| 1 | Self-pollination | 13 (S4, S9) | 13 (S4, S9) | 128 | 28 | 1 | 157 | 1,65% |
| 2 | Self-pollination | 13 (S4, S9) | 13 (S4, S9) | 104 | 10 | 1 | 115 |  |
| 3 | Self-pollination | 13 (S4, S9) | 13 (S4, S9) | 60 | 20 | 4 | 84 |  |
| 4 | Self-pollination | 13 (S4, S9) | 13 (S4, S9) | 76 | 10 | 1 | 87 |  |
| 5 | Self-pollination | 13 (S4, S9) | 13 (S4, S9) | 12 | 2 | 1 | 15 |  |
| 6 | Self-pollination | 13 (S4, S9) | 13 (S4, S9) | 20 | 6 | 0 | 26 |  |
| 1 | Incompatible | 12 (S4, S9) | 13 (S4, S9) | 60 | 10 | 1 | 71 | 1,79% |
| 2 | Incompatible | 12 (S4, S9) | 13 (S4, S9) | 40 | 6 | 1 | 47 |  |
| 3 | Incompatible | 12 (S4, S9) | 13 (S4, S9) | 32 | 6 | 0 | 38 |  |
| 4 | Incompatible | 12 (S4, S9) | 13 (S4, S9) | 100 | 20 | 3 | 123 |  |
| 5 | Incompatible | 12 (S4, S9) | 13 (S4, S9) | 28 | 0 | 3 | 31 |  |
| 6 | Incompatible | 12 (S4, S9) | 13 (S4, S9) | 100 | 18 | 1 | 119 |  |
| 7 | Incompatible | 12 (S4, S9) | 13 (S4, S9) | 28 | 4 | 0 | 32 |  |
| 8 | Incompatible | 12 (S4, S9) | 13 (S4, S9) | 36 | 6 | 0 | 42 |  |
| 1 | Self-pollination | 15 (S10, S6) | 15 (S10, S6) | 32 | 0 | 0 | 32 | 0,00% |
| 2 | Self-pollination | 15 (S10, S6) | 15 (S10, S6) | 16 | 0 | 0 | 16 |  |
| 3 | Self-pollination | 15 (S10, S6) | 15 (S10, S6) | 32 | 0 | 0 | 32 |  |
| 4 | Self-pollination | 15 (S10, S6) | 15 (S10, S6) | 32 | 0 | 0 | 32 |  |
| 5 | Self-pollination | 15 (S10, S6) | 15 (S10, S6) | 44 | 0 | 0 | 44 |  |
| 6 | Self-pollination | 15 (S10, S6) | 15 (S10, S6) | 28 | 0 | 0 | 28 |  |
| 7 | Self-pollination | 15 (S10, S6) | 15 (S10, S6) | 24 | 0 | 0 | 24 |  |
| 8 | Self-pollination | 15 (S10, S6) | 15 (S10, S6) | 36 | 0 | 0 | 36 |  |
| 9 | Self-pollination | 15 (S10, S6) | 15 (S10, S6) | 80 | 0 | 0 | 80 |  |
| 10 | Self-pollination | 15 (S10, S6) | 15 (S10, S6) | 28 | 0 | 0 | 28 |  |
| 11 | Self-pollination | 15 (S10, S6) | 15 (S10, S6) | 56 | 4 | 0 | 60 |  |
