# Supplementary material for: Gametophytic self-incompatibility in Andean capuli (Prunus serotina subsp. capuli): allelic diversity at the S-RNase locus influences normal pollen-tube formation during fertilization
Source: PeerJ. 2020 Aug 31;8:e9597. doi: 10.7717/peerj.9597 (PMC7469932; doi:10.7717/peerj.9597)

**Fig. 3** PCR amplification of the *P. serotina* C2-C3 intragenic region using the Ps1C2Fw (ATY-CAT-GGC-CTR-TGG-CCA-AG) and Ps2C3Rv (TGY-TTR-TTC-CAT-TCV-CBT-TCC) primers. Black arrows indicate the position of the expected alleles whereas white arrows highlight the unexpected alleles obtained for H25, Azu15, Pic19, Car7, Car3 and Car12.


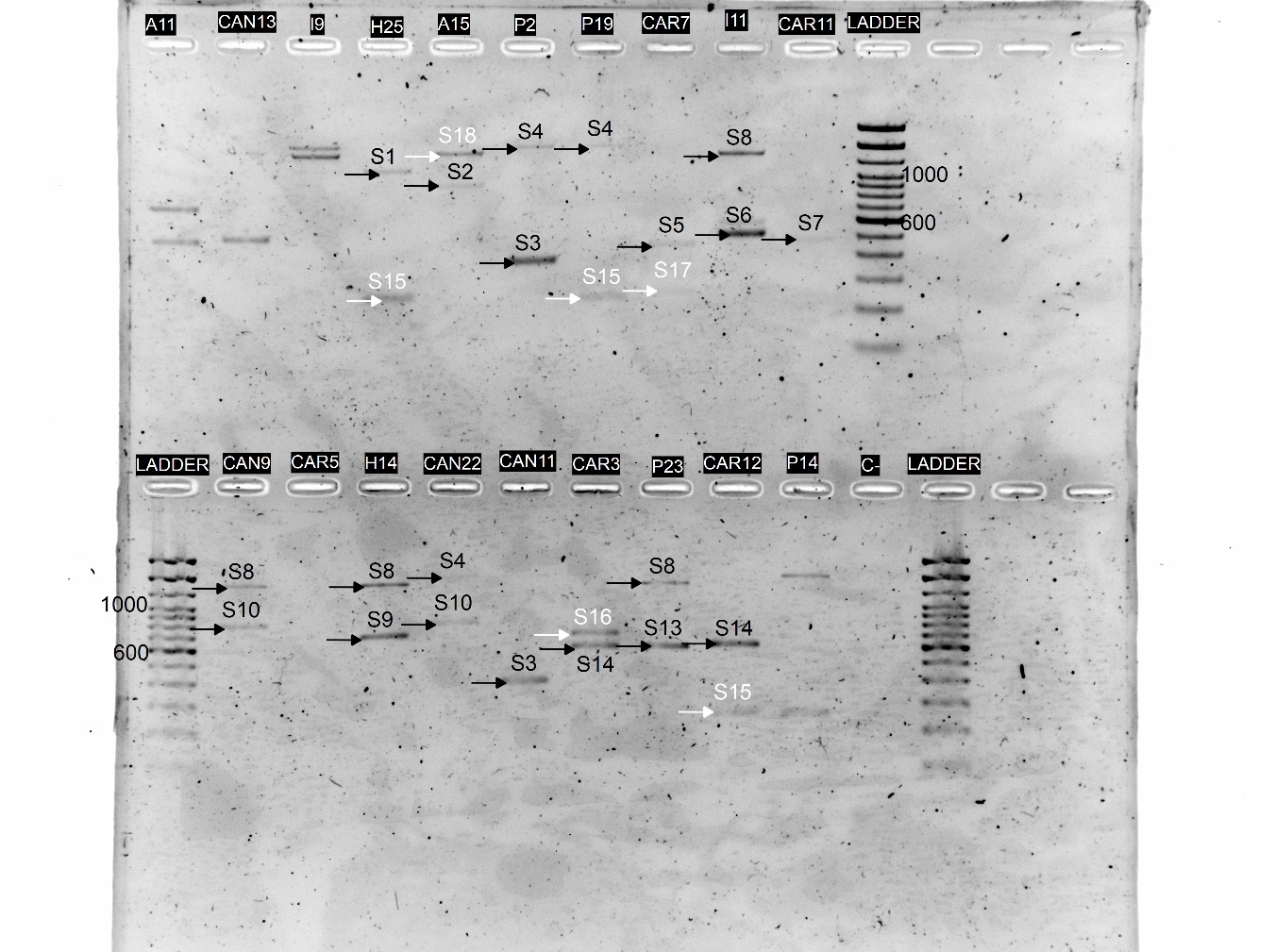


**Figure 4.** *In-vitro* CAPS patterns obtained for alleles S_1_, S_3_, S_5_, S_17_, S_18_. Letters indicate the restriction patterns reported for each enzyme in Table 1: RsaI, MboI and HinfI, respectively.


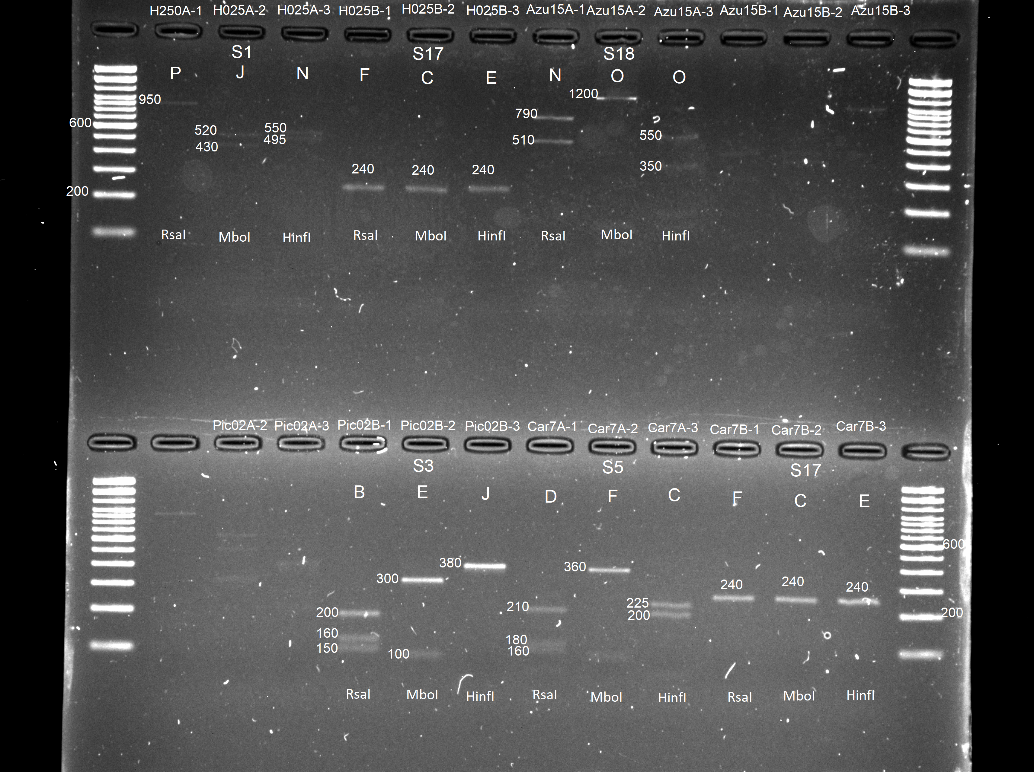


**Figure 4.** *In-vitro* CAPS patterns obtained for alleles S_4_, S_6_, S_7_, S_10_, S_12_. Letters indicate the restriction patterns reported for each enzyme in Table 1: RsaI, MboI and HinfI, respectively.


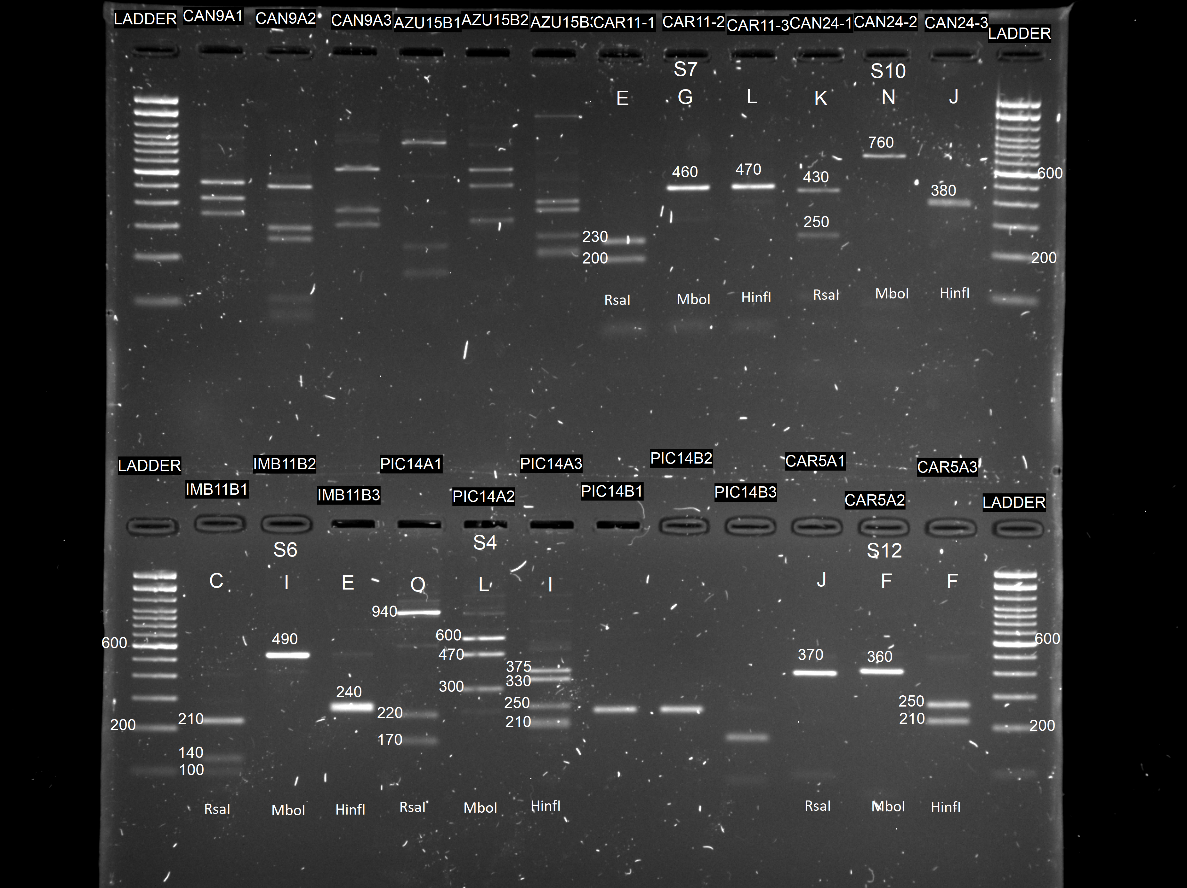


**Figure 4.** *In-vitro* CAPS patterns obtained for allele S_9_. Letters indicate the restriction patterns reported for each enzyme in Table 1: RsaI, MboI and HinfI, respectively.


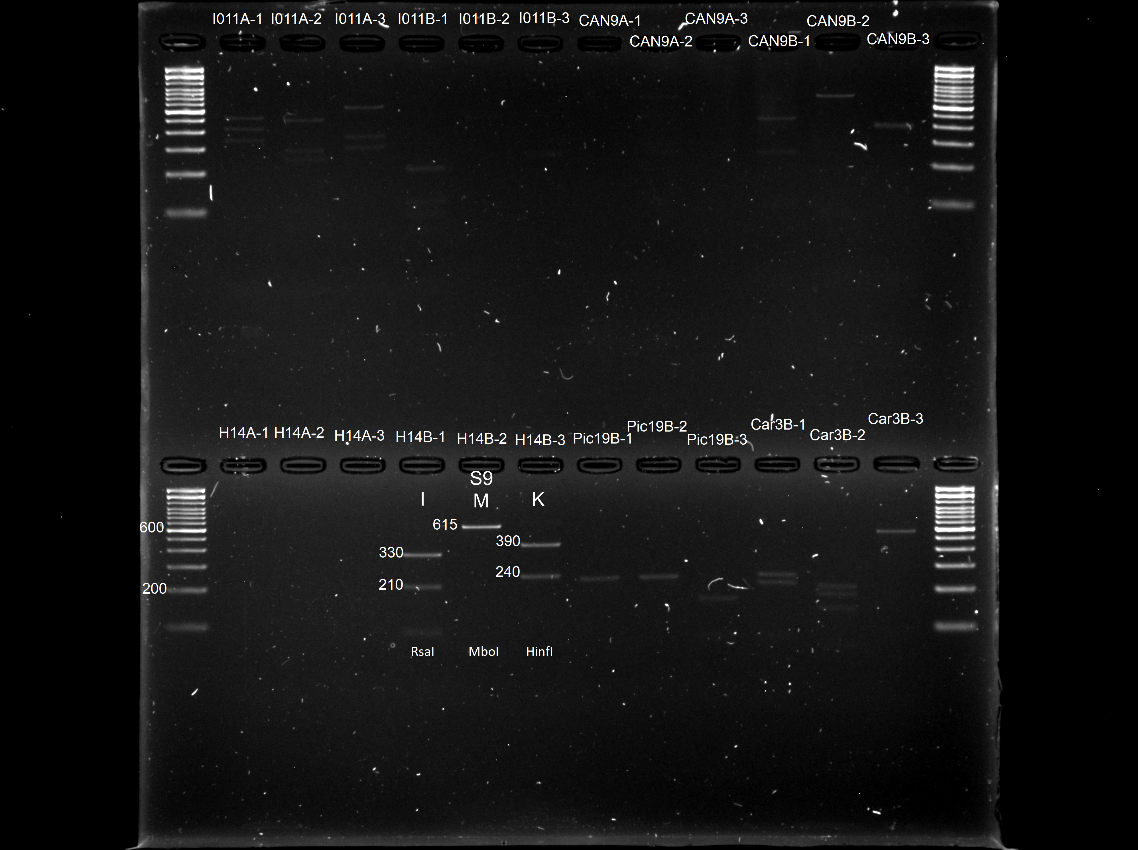


**Figure 4.** *In-vitro* CAPS patterns obtained for alleles S_8_, S_13_, S_14_, S_15_. Letters indicate the restriction patterns reported for each enzyme in Table 1: RsaI, MboI and HinfI, respectively


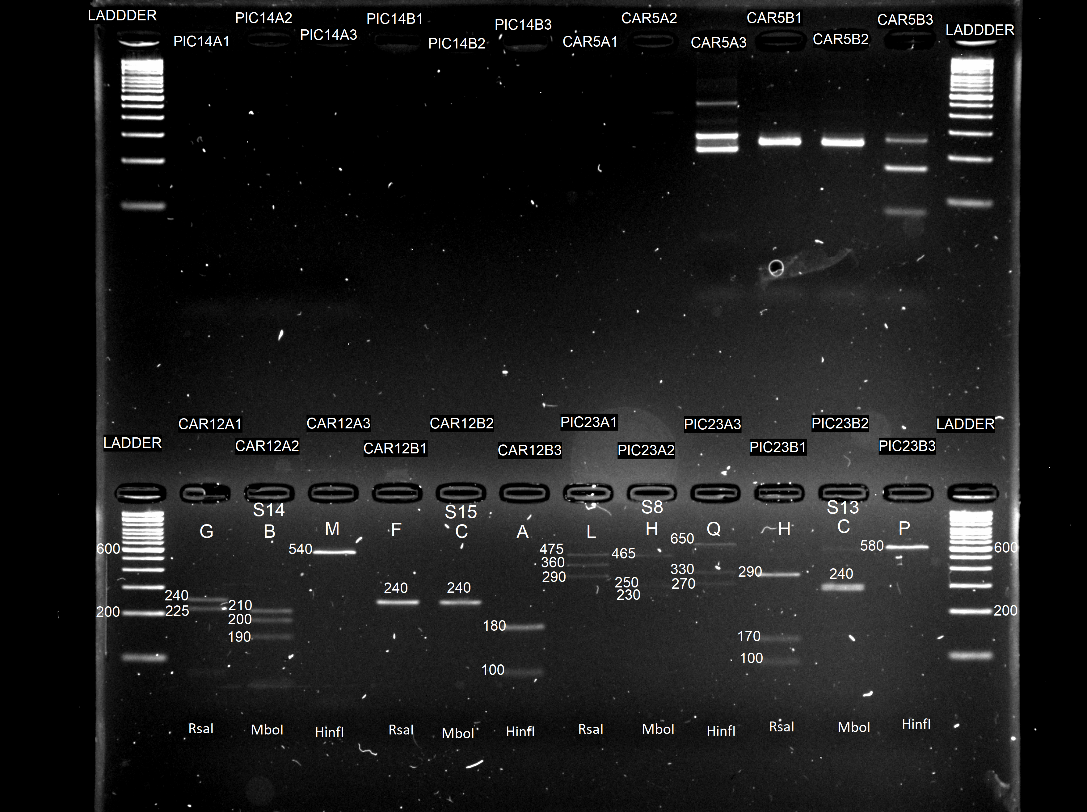


**Figure 4.** *In-vitro* CAPS patterns obtained for allele S_16_. Letters indicate the restriction patterns reported for each enzyme in Table 1: RsaI, MboI and HinfI, respectively


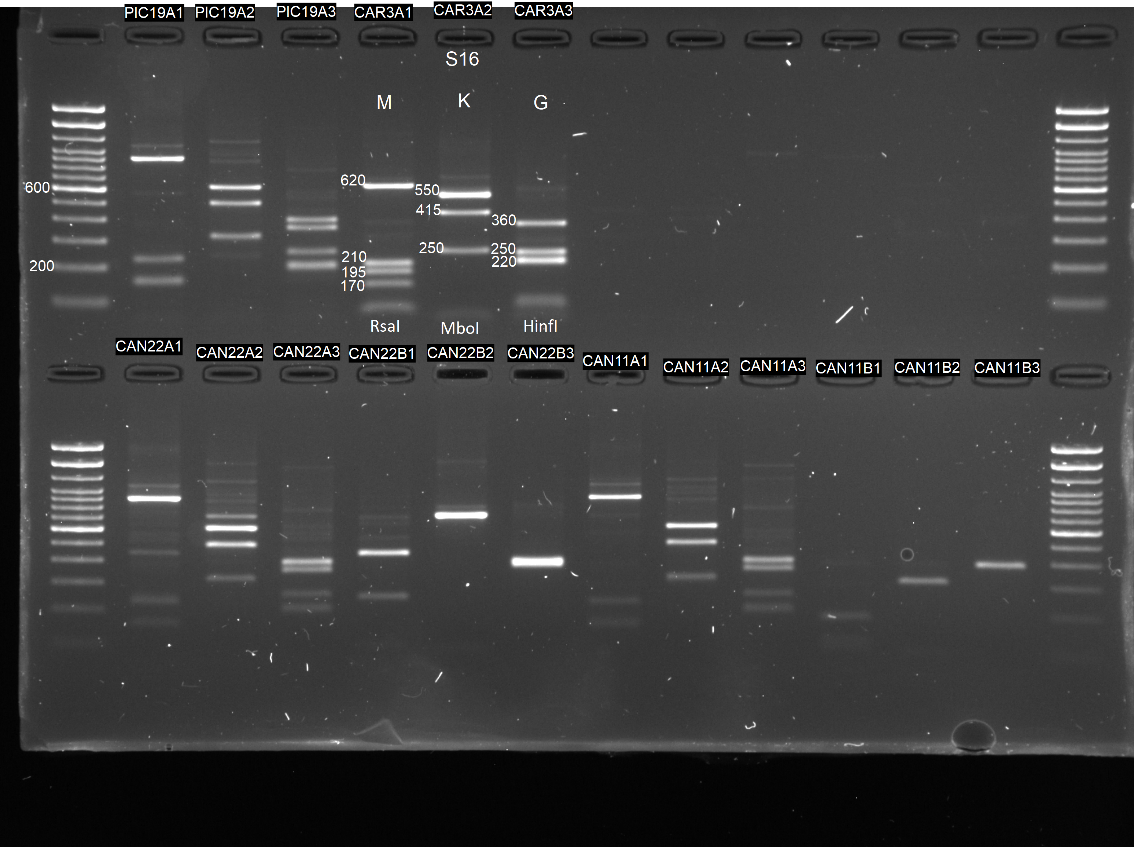

Supplement: Supplemental Information 1 [file peerj-08-9597-s006.docx]
